# Metabolome Analysis Identified Okaramines in the Soybean Rhizosphere as a Legacy of Hairy Vetch

**DOI:** 10.3389/fgene.2020.00114

**Published:** 2020-02-24

**Authors:** Nozomu Sakurai, Hossein Mardani-Korrani, Masaru Nakayasu, Kazuhiko Matsuda, Kumiko Ochiai, Masaru Kobayashi, Yusuke Tahara, Takeshi Onodera, Yuichi Aoki, Takashi Motobayashi, Masakazu Komatsuzaki, Makoto Ihara, Daisuke Shibata, Yoshiharu Fujii, Akifumi Sugiyama

**Affiliations:** ^1^Bioinformation and DDBJ Center, National Institute of Genetics, Mishima, Japan; ^2^Kazusa DNA Research Institute, Kisarazu, Japan; ^3^Department of International Environmental and Agricultural Sciences, Faculty of Agriculture, Tokyo University of Agriculture and Technology, Fuchu, Japan; ^4^Research Institute for Sustainable Humanosphere, Kyoto University, Gokasho, Japan; ^5^Department of Applied Biological Chemistry, Faculty of Agriculture, Kindai University, Nara, Japan; ^6^Division of Applied Life Sciences, Graduate School of Agriculture, Kyoto University, Kyoto, Japan; ^7^Research and Development Center for Five-Sense Devices, Kyushu University, Fukuoka, Japan; ^8^Faculty of Information Science and Electrical Engineering, Kyushu University, Fukuoka, Japan; ^9^Tohoku Medical Megabank Organization, Tohoku University, Sendai, Japan; ^10^Center for Field Agriculture Research & Education, Ibaraki University, Ami, Japan

**Keywords:** hairy vetch, untargeted metabolomics, okaramine, rhizosphere, soybean

## Abstract

Inter-organismal communications below ground, such as plant–microbe interactions in the rhizosphere, affect plant growth. Metabolites are shown to play important roles in biological communication, but there still remain a large number of metabolites in soil to be uncovered. Metabolomics, a technique for the comprehensive analysis of metabolites in samples, may uncover the molecules that intermediate these interactions. We conducted a multivariate analysis using liquid chromatography (LC)—mass spectrometry (MS)-based untargeted metabolomics in several soil samples and also targeted metabolome analysis for the identification of the candidate compounds in soil. We identified okaramine A, B, and C in the rhizosphere soil of hairy vetch. Okaramines are indole alkaloids first identified in soybean pulp (*okara*) inoculated with *Penicillium simplicissimum* AK-40 and are insecticidal. Okaramine B was detected in the rhizosphere from an open field growing hairy vetch. Okaramine B was also detected in both bulk and rhizosphere soils of soybean grown following hairy vetch, but not detected in soils of soybean without hairy vetch growth. These results suggested that okaramines might be involved in indirect defense of plants against insects. To our knowledge, this is the first report of okaramines in the natural environment. Untargeted and targeted metabolomics would be useful to uncover the chemistry of the rhizosphere.

## Introduction

Inter-organismal interactions in the rhizosphere exert a diverse range of influences on plant growth and health, and thus are pivotal in the robustness of field crops (Berendsen et al., 2012; Quiza et al., 2015; de Vries and Wallenstein, 2017). Recent studies provide evidence that plant metabolites secreted from roots shape the assemblage and functions of organisms in the rhizosphere (Stringlis et al., 2018; Huang et al., 2019; Voges et al., 2019). The rhizosphere has one of the highest densities of microbes on Earth (Reinhold-Hurek et al., 2015), and thus accumulates a wide variety of metabolites from both plants and other organisms. Fungi are well known to produce and secrete highly bioactive metabolites, some of which are used as antibiotics or other pharmaceuticals (Hautbergue et al., 2018; Helaly et al., 2018). Plants and fungi synthesize a diverse range of bioactive compounds with functions in symbiosis, defense, attraction, and repellence. Most of these metabolites are associated with specific taxa, possibly because they confer adaptive advantages during evolution (Pichersky and Lewinsohn, 2011). Despite accumulating knowledge of specialized metabolites in the rhizosphere, a tremendous number remain to be characterized.

Metabolomics—a technology for the comprehensive detection of metabolites in samples (Hall, 2006)—may uncover the nature of chemicals in the rhizosphere. Untargeted metabolomic approaches that obtain an overview of metabolite profiles including unknown compounds are used for the discovery of the candidates of characteristic metabolites to the specific samples. Targeted metabolomics approaches that accurately detect specific metabolites are then adopted for the identification and quantification of the specific compounds. Metabolomics has been employed to unravel plant-microbe interactions. For example, metabolomics analysis revealed the abundance of phenylpropanoids in root exudates of *Arabidopsis thaliana* which accommodates phenylpropanoid-utilizing microbes in the rhizosphere (Narasimhan et al., 2003). Targeted metabolomic analysis using LC-MS suggested that the infection of a plant bacterial pathogen *Pseudomonas syringae* pv. tomato (Pst) DC3000 to *A. thaliana* reprograms plant signaling and primary metabolic pathways to modulate stomatal movement (Pang et al., 2018). Recently, untargeted metabolite profiling of *Eucalyptus grandis* seedlings infected with *Armillaria luteobuablina*, a necrotrophic pathogen, identified threitol as a key metabolite to promote the infection (Wong et al., 2019). So far, specialized metabolites secreted from roots have traditionally been extracted from hydroponically grown axenic plants for metabolomics analysis. However, to gain insights into the nature of compounds of both plant and fungal origins and to elucidate their functions in field conditions, comprehensive metabolomic analysis of the rhizosphere is informative (van Dam and Bouwmeester, 2016).

Here, we report for the first time the identification of okaramines in the rhizosphere of hairy vetch (*Vicia villosa* Roth subsp. *villosa*) using untargeted and targeted metabolome analyses. Okaramines were first identified in soybean pulp (*okara*) inoculated with *Penicillium simplicissimum* AK-40 (Hayashi et al., 1989), and shown to have pesticidal activities. Biosynthesis pathway for okaramines in *P. simplicissimum* have been revealed. Okaramines are synthesized from L-Trp and dimethylallyl pyrophosphate *via* condensation of two tryptophan molecules by nonribosomal peptide synthetase (NRPS), followed by the prenylation by dimethylallyl tryptophan synthase (DMATS) (Kato et al., 2018). *P. simplicissimum* has a gene cluster for okaramine biosynthesis, and okaramine B, with the highest bioactivity among okaramines (Furutani et al., 2014; Furutani et al., 2017; Kato et al., 2018), is the final product. Hairy vetch is used as a cover crop or a green manure crop in Japanese orchards and rice fields because of its many merits, from weed control to suppression of bacterial and fungal diseases (Fujii and Appiah, 2019). Cyanamide was identified from the rhizosphere of hairy vetch as an allelochemical (Kamo et al., 2003; Kamo et al., 2008), but other metabolites that contribute the rhizosphere interactions remain unknown. We have maintained a field of hairy vetch and soybean (*Glycine max*) rotation system for 11 years. We detected okaramines in the rhizosphere of soybean in this rotation system at concentrations where they can exhibit insecticidal activity (Hayashi et al., 1989; Furutani et al., 2014; Kato et al., 2018), but did not detect them in soybean field without previous hairy vetch growth. Possible functions of okaramines in the rhizosphere are discussed.

## Materials and Methods

### Chemicals

Authentic standards of okaramines A and C were provided by Dr. Hayashi (Hayashi et al., 1989; Hayashi et al., 1991a). We prepared okaramine B for this study as described previously (Hayashi et al., 1989; Hayashi et al., 1991b). In brief, *okara* was fermented with *P. simplicissimum* AK-40 and extracted with acetone. The extract was concentrated *in vacuo* and partitioned with ethyl acetate and water. The ethyl acetate layer was concentrated *in vacuo* and the residue was purified by silica gel column chromatography with hexane–ethyl acetate to yield okaramine B. The structures were confirmed by ^1^H- and ^13^C-NMR (**Supplementary Figure 1**). Methanol, acetonitrile, formic acid, and ultrapure water for the extraction and LC-MS analysis were obtained from Wako Pure Chemical Industries (Osaka, Japan) or Nacalai Tesque (Kyoto, Japan), unless otherwise stated.

### Plant Materials and Soils

We used the following soils: TUAT-RH and TUAT-RZ, collected from the rhizosphere (RH) and the root zone (RZ) in pots where hairy vetch had been grown on a commercial plant culture soil (Kumiai Nippi Engei Baido No. 1, Zen-Noh Co, Tokyo, Japan) at Tokyo University of Agriculture and Technology (TUAT) for 4 weeks; After 4 weeks, plants were gently removed from the pots and placed on a clean plastic sheet. Roots were then shaken vigorously to collect root zone soil (TUAT-RZ). Then, the soil adhered to the roots was gently brushed off with a clean brush to collect the rhizosphere soil on clean paper (TUAT-RH). The latter soil was passed through a 2-mm × 2-mm mesh to remove large organic material and gravel. Kazusa, a mixture of equal volumes of vermiculite (10865595 SK Agri K.K., Gunma, Japan) and a plant culture soil (New Power Soil, Kureha Corporation, Tokyo, Japan); and KUAS-F and KUAS-R, a gray lowland soil (Sugiyama et al., 2014) collected after the growth of soybean in the field (F) or in a rhizobox (R) at the Kyoto University of Advanced Science (KUAS).

For the field experiment, seeds were planted in the field at TUAT in November, and plants were allowed to grow to 20 cm tall (~3 weeks). Forty healthy plants were dug out with a shovel and placed into a plastic bag in a cool box containing dry ice. The rhizosphere soil was collected as described above in the laboratory.

We planted soybean (*Glycine max* cv. Enrei) in an experimental field of Ibaraki University, in Ibaraki prefecture, where hairy vetch had been grown as a cover crop for 11 years. We harvested six healthy plants and collected both the soybean rhizosphere soil and bulk soil.

In all experiments, bulk soil was collected below the top 5 cm.

Hairy vetch seeds were sterilized by soaking in 70% (v/v) ethanol for 10 seconds and 1% (v/v) sodium hypochlorite for 5 min. Seeds were washed 3 times with sterile distilled water. Hairy vetch was grown in a 200 ml beaker containing 60 ml water agar medium. Seedlings were kept in an incubator (16 h light 8 h dark) at 25°C for 10 days. The seedlings were then harvested and kept in -80°C until metabolite extraction. Both aerial part and roots (30 mg) were extracted three times with 200 µl of methanol, vortexed, sonicated for 15 min, and centrifuged at 20,000 × *g* for 5 min. The supernatants were collected, filtered through a Minisart 0.45-μm filter (Sartorius, Göttingen, Germany), and analyzed by LC-MS. Hairy vetch was also grown hydroponically in water with aeration using an air pump (e-AIR 1000SB, GEX Co. Ltd., Japan) and without addition of any nutrient in an incubator (16 h light 8 h dark) at 25°C. The hydroponic water was collected as root exudates from seedlings at 15 days after germination. Root exudates were lyophilized and reconstituted in methanol for LC-MS analysis.

### Untargeted Metabolome Analysis

Frozen soil samples (300 mg) were extracted with 900 µl of methanol containing 25 µM 7-hydroxy-5-methylflavone (EXTRASYNTHESE, Lyon, France) as an internal standard. The samples were mixed twice for 2 min each in a Mixer Mill MM 300 (Qiagen K.K., Tokyo, Japan) at 25 Hz, and the homogenates were centrifuged (17,400 × *g* for 5 min at 4°C). The supernatant was filtered through a 0.2-µm polytetrafluoroethylene membrane (Millipore), and hydrophobic compounds in the filtrate were removed by adsorption to a C18 silica column (MonoSpin C18, GL Science, Tokyo, Japan). The eluate was used for the untargeted metabolome analysis by liquid chromatography (LC)—mass spectrometry (MS). Blank samples were prepared without soil.

The untargeted metabolome analysis was performed on an Agilent 1100 system (Agilent, Palo Alto, CA, USA) coupled to a Finnigan LTQ-FT (Thermo Fisher Scientific, Waltham, MA, USA). An aliquot (20 µl) of the methanol solution was applied to a TSK-gel column (ODS-100V, 4.6 × 250 mm, 5 µm; Tosoh Corporation, Tokyo, Japan). Water (HPLC grade; solvent A) and acetonitrile (HPLC grade; solvent B) were used as the mobile phase with 0.1% *v*/*v* formic acid in both. The gradient program was as follows: 3% B (0 min), 97% B (90 min), 97% B (100 min), 3% B (100.1 min) and 3% B (107 min). The flow rate was 0.25 ml/min for 0–100.1 min and 0.5 ml/min for 100.1–107 min. The column oven temperature was set to 40°C. The mass range was *m/z* 100–1500. The electrospray-ionization (ESI) settings were a spray voltage of 4.0 kV and a capillary temperature of 300°C. The nitrogen sheath gas and auxiliary gas were set at 40 and 15 arbitrary units, respectively. Multistage MS (MS*^n^*) analysis was performed in ESI positive mode as follows: full mass scan with Fourier Transform Ion Cyclotron Resonance at a resolution of 100,000, and MS^2^ scans by collision-induced dissociation for the five most intense ions of the full mass scan in ion trap mode. The HPLC eluate was monitored with a photodiode array detector with a wavelength range of 200–650 nm. Data were acquired and browsed in Xcalibur v. 2.0.7 software (Thermo Fisher Scientific), once for each extraction from a sample. The blank sample was analyzed in triplicate. The binary raw data from Xcalibur (.raw) and the experimental metadata for the samples are available on our website (http://webs2.kazusa.or.jp/data/okaramine) and on Metabolonote (http://metabolonote.kazusa.or.jp/SE194:/) (Ara et al., 2015), respectively.

The raw data generated by Xcalibur software were converted to mzXML format with the MSConvert function of ProteoWizard v. 3.0.7155 software (Chambers et al., 2012) and processed in PowerGetBatch software (Sakurai and Shibata, 2017) for peak detection, characterization, and alignment. We selected the metabolite peaks that were detected in at least one soil sample and not detected in the blank samples for further data processing. The detailed parameters for PowerGetBatch are available on our website (http://webs2.kazusa.or.jp/data/okaramine). The peaks were annotated based on the compound database search using the UC2 database at the MFSearcher website (Sakurai et al., 2018) with the predicted mass values of the original molecules at a given 5-ppm mass tolerance, and the KNApSAcK (Afendi et al., 2012), KEGG (Kanehisa et al., 2016), HMDB (Wishart et al., 2013), LIPID MAPS (Fahy et al., 2009), and flavonoid (http://metabolomics.jp/wiki/Category : FL) databases. The images of two-dimensional mass chromatograms were generated by MassChroViewer software (Sakurai and Shibata, 2017) using the mzXML files. A value of 1/32 of the maximum ion intensity in the mass chromatogram was set as the maximum color strength of the image.

Principal component analysis (PCA) was performed in SIMCA v. 14 software (Sartorius Stedim Biotech, Göttingen, Germany). The peak intensity values estimated by PowerGetBatch were log_10_-transformed and centered on the median value in the sample. Missing values were filled with 1/10 of the smallest peak intensity among all samples. The values for the aligned metabolite peak were further scaled to unit variance scaling. Other statistical analyses were performed in Excel software (Microsoft).

### LC-IT-TOF-MS Analysis for Identification of Okaramines

The authentic standards of okaramines A, B, and C, and soil extracts prepared as described above were analyzed by LC-IT-TOF-MS (Shimadzu, Kyoto, Japan). The samples (5 µl) were injected to a TSKgel ODS-80Ts column (5.0 µm, 2.0 × 250 mm, Tosoh Corporation, Tokyo, Japan). The column oven was set at 40°C and the flow rate was 0.2 ml/min. The mobile phases were water containing 0.1% (*v/v*) formic acid (solvent A) and acetonitrile containing 0.1% (*v/v*) formic acid (solvent B) with gradient elution of 3%–97% B at 0–72 min (linear), 97% B at 72–84 min (linear), 97% B at 84–96 min, 3%–97% B at 96–116 min (linear), 97% B at 116–128 min, and 3% B at 128–148 min. MS conditions were as follows: ionization, ESI; probe voltage, + 4.5 kV and – 3.5 kV; CDL temperature, 200°C; heating block temperature, 200°C; nebulizing gas flow, 1.5 L/min; drying gas pressure, 100 kPa. MS scan was in the range of *m/z* 520–570. MS/MS scan was in the range of *m/z* 100–570 for the most intense ion of the MS scan. Data acquisition and analysis were performed using LCMS solution software (Shimadzu, Kyoto, Japan).

### MRM Using LC-Triple Quadrupole-MS for Quantification of Okaramines

Soil samples (300 mg) were extracted with 300 µl of methanol, vortexed, sonicated for 15 min, and centrifuged at 20,000 × *g* for 5 min. The supernatants were collected, and the residues were further extracted twice with 300 µl methanol. The bulked supernatants were purified through a Sep-Pak C18 Plus short cartridge (Waters, Milford, MA, USA). The eluate was filtered through a Minisart 0.45-μm filter (Sartorius, Göttingen, Germany). The filtrate (300 µl) was dried *in vacuo*, dissolved in 30 µl of methanol, and analyzed by LC-MS.

LC-MS analysis was performed on an Acquity UPLC H-Class/Xevo TQD (Waters). LC conditions were as follows: column, Acquity UPLC BEH C-18 (1.7 µm, 2.1 × 50 mm, Waters) with UPLC BEH C18 VanGuard Pre-column (1.7 µm, 2.1 × 5 mm), 40°C; injection volume, 2 µl; flow rate, 0.2 ml/min; mobile phases, water containing 0.1% (*v/v*) formic acid (solvent A) and acetonitrile (solvent B) with gradient elution of 10%–50% B at 0–5 min (linear), 50%–70% B at 5–15 min (linear), 100% B at 15–19 min, and 10% B at 19–24 min. MS conditions were as follows: ionization, positive ESI; capillary voltage, 3.15 kV; source temperature, 150°C; desolvation gas temperature, 400°C; nebulizer and desolvation N_2_ gas flow rates, 50 and 800 L/h, respectively. The authentic standards of okaramine A, B, and C were monitored by MS scan with a cone voltage of 60 V in the range of *m/z* 400**–**600 and MS/MS scan with collision energies of 15 and 30 eV in the range of *m/z* 100**–**580 for precursor ions of *m/z* 521.5, 567.6, and 525.5 for okaramines A, B, and C, respectively. Okaramines from soil samples were detected by multiple reaction monitoring (MRM) under the following conditions: transition, *m/z* 521.4 > 453.4 for okaramine A, *m/z* 567.4 > 475.4 for okaramine B, and *m/z* 525.4 > 457.4 for okaramine C; cone voltage, 30 V; collision energy, 15 eV for okaramines A and C, 30 eV for okaramine B; monitoring time, 8.65–9.50 min for okaramine B, 9.40–10.25 min for okaramine A, and 10.20–11.05 min for okaramine C. Data were analyzed in MassLynx v. 4.1 software (Waters). Quantities of okaramines were calculated from the peak area by using calibration curves established by the analysis of authentic okaramines.

### Taste Sensor Measurements for Soil Samples

Soil samples were analyzed by taste sensors (Toko et al., 2016) based on chemical interactions to lipid polymer membranes (Tahara and Toko, 2013). Details are described in **Supplementary Methods**.

### Measurement of Soil Minerals

Contents of nutrients in rhizosphere soils were determined by a protocol scaled down from standard soil analysis methods because the rhizosphere soil samples were available only in small quantities. Details are described in **Supplementary Methods**.

## Results

### Untargeted Metabolome Analysis Depicting Chemical Diversity of the Soils

We conducted an untargeted metabolome analysis for five different soil samples using reverse-phase LC-MS. In ESI positive mode we detected 145 peaks in TUAT-RH, 100 in TUAT-RZ, 214 in Kazusa, 336 in KUAS-F, and 244 in KUAS-R. In total, 474 unique compound peaks were recognized after alignment (**Supplementary Table 1**). The soils had fewer peaks than foods (~4,300, on average) (Sakurai and Shibata, 2017). The overview of metabolite peaks was shown in **Supplementary Figure 2** as two-dimensional mass chromatograms. The metabolite profiles were different from each other, but the rhizosphere and root zone soils from TUAT had similar profiles. The chemical similarity in TUAT soils was also observed in the result of PCA score plot (**Supplementary Figure 3A**) where the TUAT soils were plotted at similar locations. This feature of chemical similarities among the soil samples was also observed in the measurement of minerals (**Supplementary Table 2**) and taste sensors (**Supplementary Figure 4**) which can respond to a broader range of chemicals, although narrowing down the candidates based on minerals and sensors was not feasible with the limited number of samples.

### Identification of Okaramines by Comparative and Targeted Metabolomics

**Figure 1:**
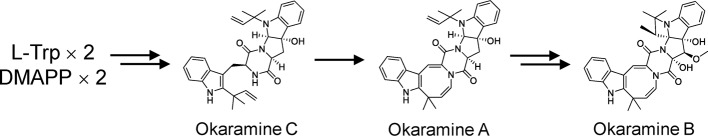
Okaramine B biosynthesis pathway in *Penicillium simplicissimum* AK-40 proposed by (Kato et al., 2018).

The metabolomic similarity between the TUAT soils mentioned above implied that TUAT soils including hairy vetch rhizosphere might contain some specific metabolites related to the rhizosphere. We focused on the metabolites which were specifically accumulated in these TUAT soils and were absent in other soils from **Supplementary Table 1** using Excel software (version 2016, Microsoft). As a result, 33 metabolite peaks were selected (**Supplementary Table 3**). On the loading plot of PCA (**Supplementary Figure 3B**), all 33 peaks were located in the same direction as that TUAT soils were located on the score plot (**Supplementary Figure 3A**, arrow). The annotation from compound database search showed a large number of potential N-containing compounds. We surveyed literature for the annotated compounds for 33 peaks and focused on one candidate “okaramines” (**Figure 1**) for further identification based on the following reasons. (1) Okaramines were identified in the medium cultured with a soil microorganism, however, occurrence in nature have not been reported (2). Their insecticidal activity might be involved in potential allelopathic functions. (3) We found no peak with similar mass values to the okaramines (at the 5-ppm mass tolerance) in the Food Metabolome Repository (Sakurai and Shibata, 2017) which includes untargeted metabolome data from various plants such as 57 vegetables and 28 fruits, consistent with the assumption that okaramines are biosynthesized in the soils. (4) The MS^2^ spectra for the okaramine A and C candidates showed fragment peak likely derived from the cleavage of the C_5_ moiety (**Supplementary Figure 5**), although the MS^2^ spectra of okaramines have not been reported to date. (5) Several okaramine derivatives including intermediates were listed in the annotations of the 33 peaks, implying the active okaramine biosynthesis in these soils. (6) the authentic standards of okaramines which were essential for identification were available.

**Figure 2:**
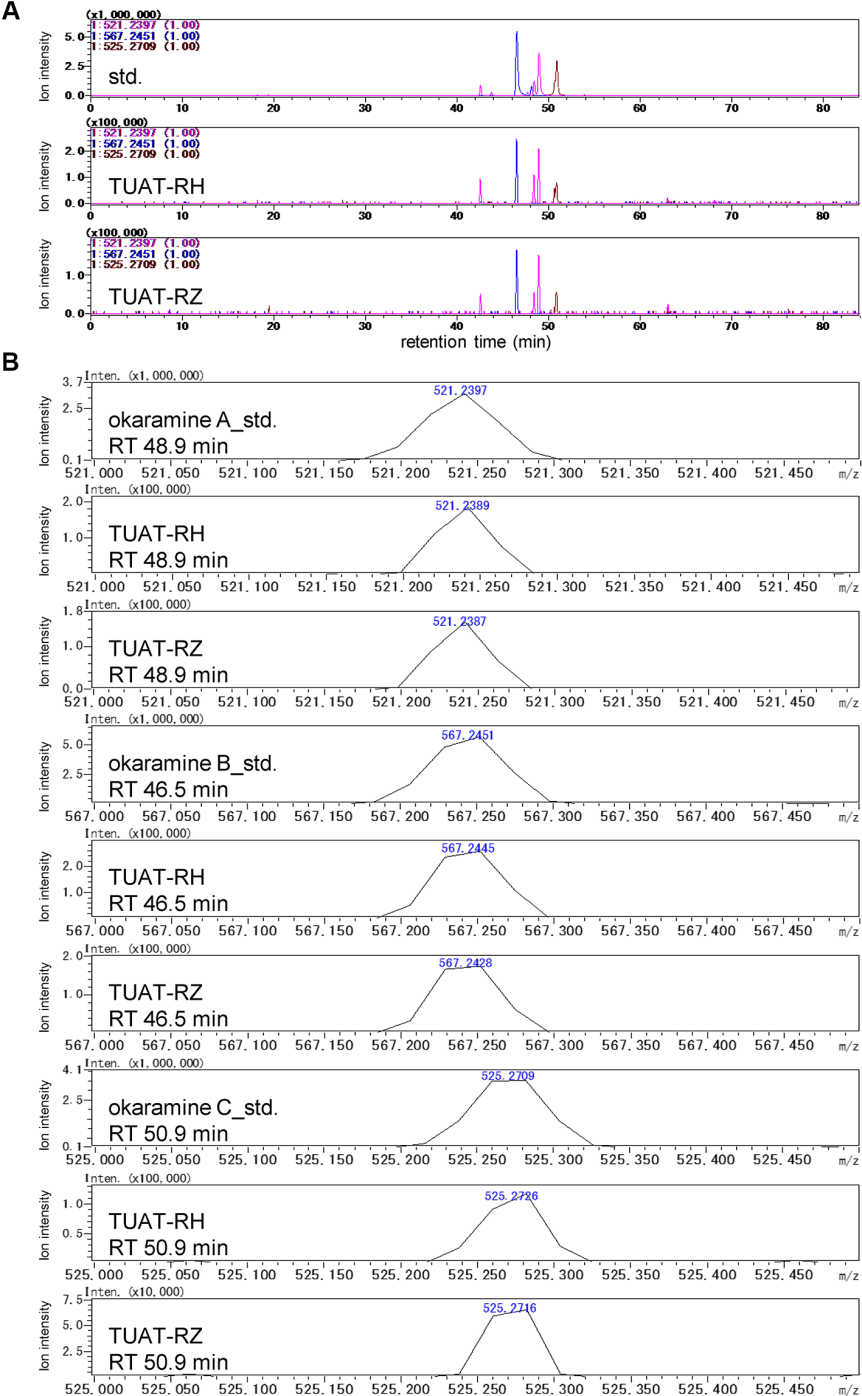
LC-MS analysis of the authentic standards of okaramines A, B, and C, and TUAT soil samples using LC-IT-TOF-MS. Extracted ion chromatograms for *m/z* 521.2397, 567.2451, and 525.2709 are shown in **(A)**. Accurate mass of peaks in **(A)** at retention times of 46.5, 48.9, and 50.9 min are shown in **(B)**.

Okaramines A, B, and C were identified in the TUAT soils by comparing the retention time and accurate mass of the candidate peaks with those from authentic standards under the same experimental condition using LC-IT-TOF-MS (Shimadzu) (**Figure 2**) because the LC-FT-ICR-MS used in the untargeted analysis was disposed before the identification of okaramines. The TUAT soils contained the peaks which correspond to the main peaks for authentic standards of okaramines A, B, and C at the same retention time on the extracted mass chromatograms (**Figure 2A**). The mass values of the candidate peaks were identical to those of the authentic standards within 5-ppm (**Figure 2B**). The confidence of the identification corresponded to “level 1” according to the guideline of Metabolomics Standards Initiative (Sumner et al., 2007). Although the MS/MS spectra of okaramines in soil samples were not obtained due to the limitation of the instrument, the MS/MS spectra of authentic standards were comparable to those obtained in soil samples analyzed in LC-FT-ICR-MS, especially for the neutral mass loss (“68”) of the product ion peak attributable to the fragmentation of the C_5_ structure in okaramines A and C, and for the fragment peak 198 attributed to a molecular ion which would be specifically generated from okaramine C (**Supplementary Figure 6**). A targeted metabolome analysis by multiple reaction monitoring (MRM) analysis using LC-triple quadrupole-MS (Waters) was conducted to quantify the okaramines at a high selectivity and sensitivity. The extracted mass chromatograms for the authentic standards of okaramines showed similar profiles to those obtained by LC-IT-TOF-MS. Using the LC-triple quadrupole-MS, we obtained essentially the same profiles of the extracted mass chromatograms and the MS/MS spectra to those observed in LC-IT-TOF-MS (**Supplementary Figures 6** and **7**). Although we could not detect extracted mass chromatograms from soil samples, we detected the peaks in the soil at the same retention times of the authentic standards using MRM transitions based on the above-mentioned mass spectra for okaramines. These results strongly suggested that we specifically detected the okaramines by MRM. Okaramines A, B, and C were detected in the rhizosphere (TUAT-RH) and root zone (TUAT-RZ) soils, but not in unplanted soils (**Supplementary Figure 8**). Shoots and roots of axenically grown hairy vetch and root exudates obtained from hydroponically grown hairy vetch did not contain okaramines (data not shown).

### Occurrence of Okaramines in the Field

**Figure 3:**
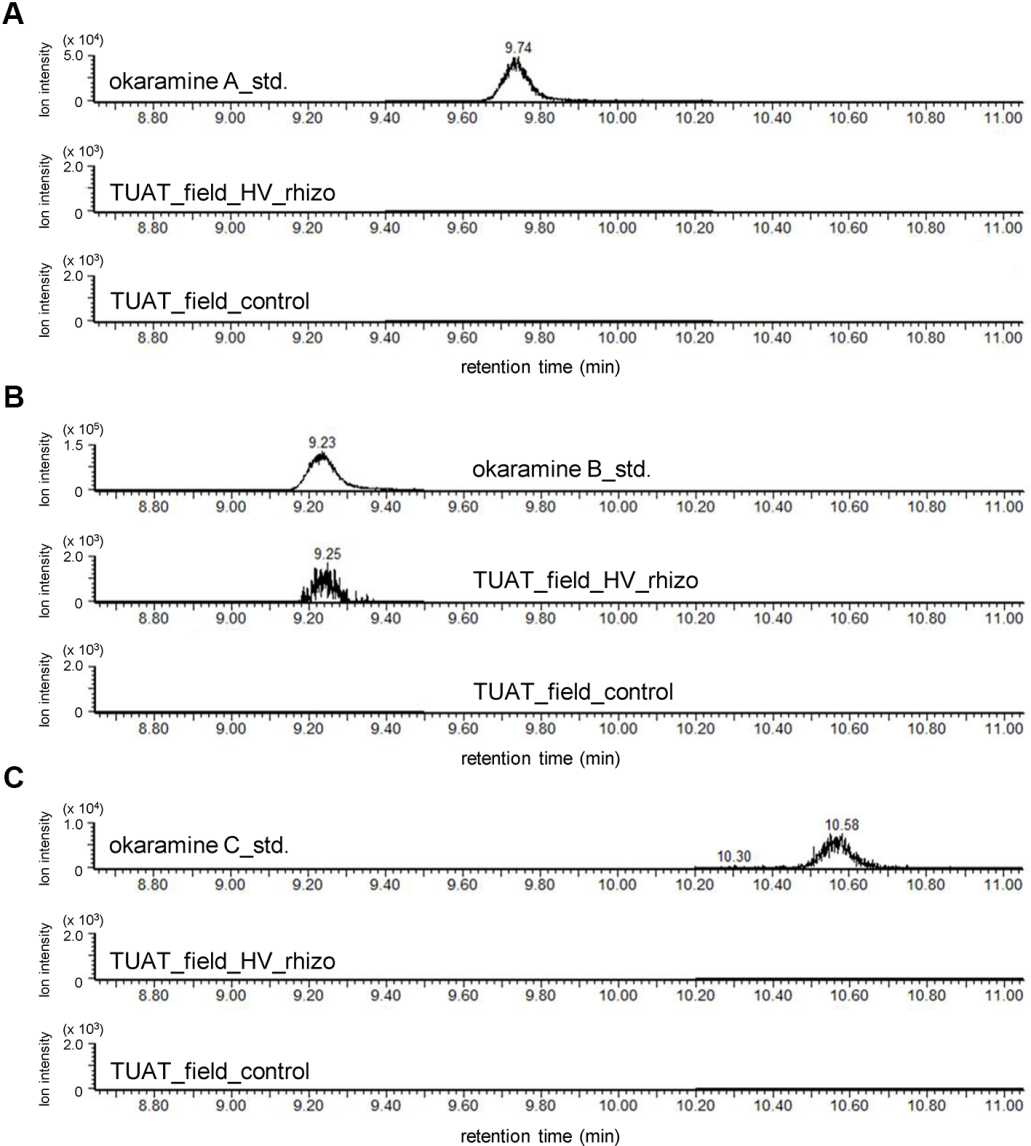
LC-MS analysis of the authentic standards of okaramines A, B, and, C, and extracts from rhizosphere soils of hairy vetch grown in TUAT field using LC-triple quadrupole-MS. Chromatograms obtained by MRM are shown for **(A**–**C)** okaramines A, B, and C. “HV_rhizo”, rhizosphere soils of hairy vetch plot; “control”, unplanted soil.

**Figure 4:**
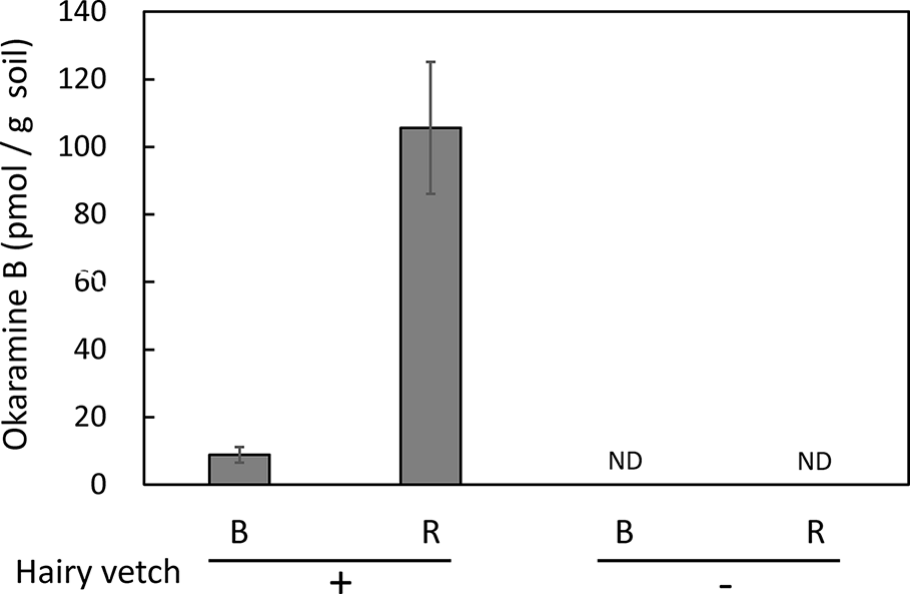
Okaramine B in soybean field soil. Okaramine contents in soybean rhizosphere and bulk soils were analyzed in LC-MS. Okaramine A and C were not detected. B, bulk soil; R, rhizosphere soil; ND, not detected. Each bar presents the mean ± SD of three biological replicates.

Untargeted and targeted metabolomics analysis revealed the presence of okaramines in rhizosphere of hairy vetch grown in pots. We then analyzed the rhizosphere soils of hairy vetch grown under fluctuating environment in field, using MRM analysis. Okaramine B (but not okaramine A or C) was detected in rhizosphere soil of hairy vetch grown in the field at TUAT, but not in the unplanted soil (**Figure 3**). Because hairy vetch is widely used in crop rotation system, we tested whether okaramines are also present in the rhizosphere of soybean grown after hairy vetch in a field, using an experimental field where hairy vetch-soybean rotation has been maintained for 11 years. Okaramine B was detected in both bulk soil and rhizosphere soil of soybean grown following hairy vetch at Ibaraki University, but not in those of soybean that did not follow hairy vetch (**Figure 4**). The concentration in the bulk soil (8.86 pmol/g) was about 92% lower than that in the rhizosphere soil (106 pmol/g). These results suggest that previous cultivation of hairy vetch is required for okaramine production in soils, and that soybean enhances the production in the rhizosphere.

## Discussion

Comparative untargeted metabolome analyses would offer a powerful way to uncover belowground chemistry. We successfully identified okaramine species, perhaps aided by the presence of fewer metabolites in soil than in plants, and found notable differences in chemical composition between soil samples. These features were advantageous for narrowing down specific peaks to TUAT soils.

Okaramines were isolated initially as insecticides from *okara* inoculated with *P. simplicissimum* AK-40 (Hayashi et al., 1989). In the insect nervous system, the γ-aminobutyric acid (GABA)-gated chloride channel and the glutamate-gated chloride channel (GluCl) are the major chloride channels responsible for inhibitory neurotransmission. Okaramines activate GluCl, which is expressed only in invertebrates (Furutani et al., 2014), and are highly selective insecticides. Since the discovery of okaramines in growth media in 1989, their occurrence in nature remained unknown. Here, through the use of untargeted metabolomics, we have for the first time found okaramines A, B, and C in soils in which hairy vetch grew. We found okaramine B from the rhizosphere soils of field grown soybean at 106 pmol/g (wet soil) comparable to a concentration with insecticidal activity (180 pmol/g diet) (Hayashi et al., 1989). Okaramines are synthesized from l-Trp and dimethylallyl pyrophosphate *via* condensation of two tryptophan molecules by non-ribosomal peptide synthetase, followed by prenylation by dimethylallyl tryptophan synthase (Kato et al., 2018). *P. simplicissimum* has a gene cluster for okaramine biosynthesis, and okaramine B, with the highest bioactivity (Furutani et al., 2014; Furutani et al., 2017; Kato et al., 2018), is the final product. The detection of okaramines A, B, and C in potting soil in which hairy vetch grew and their absence in shoot, root and root exudates, suggest their active production by hairy vetch–associated *P. simplicissimum* or related filamentous fungi. Alternatively, precursors may be converted by other rhizosphere microbes to okaramine B. In addition, the detection of only okaramine B in the rhizosphere soil of field-grown hairy vetch indicates that microbes responsible for biosynthesis in the field soil might be more abundant or more active than those in the potting soil.

In the field, okaramine B was detected in both the rhizosphere and bulk soil of a soybean crop grown after hairy vetch, but not in the same field without hairy vetch as a pre-crop. These results suggest that hairy vetch provides not only N and other nutrients, but also bioactive chemicals through associated microorganisms to the subsequent crops. Recent studies have shown that plants can recruit beneficial microbes upon pathogen attack to generate microbiomes that enable the next generation of plants to suppress attack by pathogens, insects or nematodes (Bakker et al., 2018; Berendsen et al., 2018). This “soilborne legacy” is at least in part mediated by root or seed exudates (Yuan et al., 2018). Our results support the notion that hairy vetch provides okaramines for subsequent plants to suppress damage by insects; thus, this phenomenon can be regarded as an interspecies soilborne legacy, or the indirect defense of plants against pests through the use of plant-associated microorganisms (Matsuda, 2018).

Hairy vetch has various merits such as weed control, N fixation, soil erosion control, promotion of soil porosity, and support of carnivorous ladybugs to reduce populations of harmful insects (Fujii and Appiah, 2019). It also suppresses fungal or bacterial diseases (Fujii, 2001; Horimoto et al., 2002). However, the mechanism of such actions was unknown. Cyanamide was identified from hairy vetch as an allelochemical (Kamo et al., 2003; Kamo et al., 2008), but in soil it is degraded into ammonium- or nitrate-N through urea and lost. We therefore postulate that okaramines are responsible for the control of fungal and bacterial diseases. Hairy vetch is used as pre-crop not only for soybean but also for other crops. Further detailed investigations using other post-crops will be required to understand in detail the effects of okaramines and also to harness such soil-borne legacy to improve the quality and quantity of various crops.

## Data Availability Statement

The datasets generated for this study can be found in the http://metabolonote.kazusa.or.jp/SE194:/.

## Author Contributions

Conceived and designed the experiments: NS, AS. Performed the experiments: NS, HK, MN, KO, MKob, TM, MKom, YF, MI, AS. Analyzed the data: NS, HK, MN, KM, KO, MKob, YT, TO, YA, TM, DS, YF, AS. Wrote the paper: NS, HK, MN, KM, MKob, YT, YF, AS, with input from all authors.

## Funding

This study was supported in part by grants from JST-CREST (JPMJCR17O2), from the Research Institute for Sustainable Humanosphere (Mission 5-1), and the Research Unit for Development of Global Sustainability, Kyoto University. KM was supported by KAKENHI from Japan Society for the Promotion of Science (17H01472).

## Conflict of Interest

The authors declare that the research was conducted in the absence of any commercial or financial relationships that could be construed as a potential conflict of interest.
