# Supplementary material for: Metabolome Analysis Identified Okaramines in the Soybean Rhizosphere as a Legacy of Hairy Vetch
Source: Front Genet. 2020 Feb 24;11:114. doi: 10.3389/fgene.2020.00114 (PMC7049541; doi:10.3389/fgene.2020.00114)
Supplement: Supplementary file 1 [file DataSheet_1.pdf]

Supplementary Material

1 Supplementary Methods

Taste sensor measurements for soil samples

Soil samples (50 g) were mixed with 250 mL sterile water and stirred for 30 min at room temperature, then centrifuged at 5000× *g* for 10 min. Supernatants were immediately frozen and sent to Intelligent Sensor Technology, Inc. (Kanagawa, Japan), for analysis with taste sensors (AAE for umami, CT0 for salty, CA0 for sour, C00 for bitter, AE1 for astringent). These taste sensors are used by the food industry for evaluating taste (Toko et al., 2016). They use lipid polymer membranes that respond to the physical and chemical characteristics of substances via electrostatic and hydrophobic interactions (Tahara and Toko, 2013), and are thus useful for soil discrimination based on differences in chemicals.

Measurement of soil minerals

Contents of nutrients in rhizosphere soils were determined by a protocol scaled down from standard soil analysis methods because the rhizosphere soil samples were available only in small quantities. As the samples consisted of fine particles that could be easily homogenized, there was no concern of bias. Air-dried soil samples were passed through a 0.5-mm mesh stainless steel sieve. Total C and N contents were determined in 40 mg of soil by NC analyzer (Sumigraph NC-22F, Sumika Chemical Analysis Service, Ltd., Osaka, Japan). Inorganic N was extracted by suspending 30 mg of soil in 300 µL of 0.5 M K_2SO_4, shaking for 1 h at room temperature, and centrifuging at 13 000× *g* for 10 min. Ammonium-N in the supernatant was determined with a simplified indophenol blue method (Arakawa et al., 2003) adjusted to a microplate format. In brief, 50 µL of supernatant was transferred to a well of a 96-well microplate, to which was added 20 µL of a solution containing 0.212 M potassium sodium tartrate, 0.136 M trisodium citrate, and 4 mM HCl. The well then received 40 µL of a 2:1:3 mixture of 20 mM boric acid / 0.4 M NaOH – 0.313 M 2-phenylphenol sodium salt – 1.0 mM sodium pentacyanonitrosylferrate (III). The blue color was developed by adding 90 µL of sodium hypochlorite solution containing 0.025%–0.040% active Cl with incubation at 37 °C for 20 min, then the absorbance was measured at 655 nm on a microplate reader. Nitrate-N was determined colorimetrically by the modified Cataldo method (Nishiwaki et al., 1994): 50 µL of the supernatant was transferred to a well of a 96-well microplate and dried in an oven at 70 °C. The crystals in the wells were overlaid with 10 µL of 0.05 g/mL salicylic acid and left at 80 °C for 20 min. Then 250 µL of 2 M NaOH was added to the well, and the crystals were dissolved completely by pipetting. After the reaction cooled for 20 min, the absorbance was measured at 410 nm on a microplate reader.

Exchangeable bases were extracted by suspending 30 mg of soil in 600 µL of 1 M ammonium acetate (pH 7.0), shaking for 1 h at room temperature, and centrifuging at 13 000× *g* for 10 min. The contents of Ca, Mg, and K in the supernatant were determined by atomic absorption spectrometer (AA-6200; Shimadzu, Kyoto, Japan). The precipitate was suspended in 1 mL of 80% (*v/v*) ethanol, vortexed, and centrifuged. This operation was repeated another three times. The final precipitate was resuspended in 600 µL of 2 M KCl, shaken for 1 h, and centrifuged. Ammonium-N in the supernatant was

determined by the simplified indophenol blue method described above to quantify the cation exchange capacity.

2 Supplementary Figures

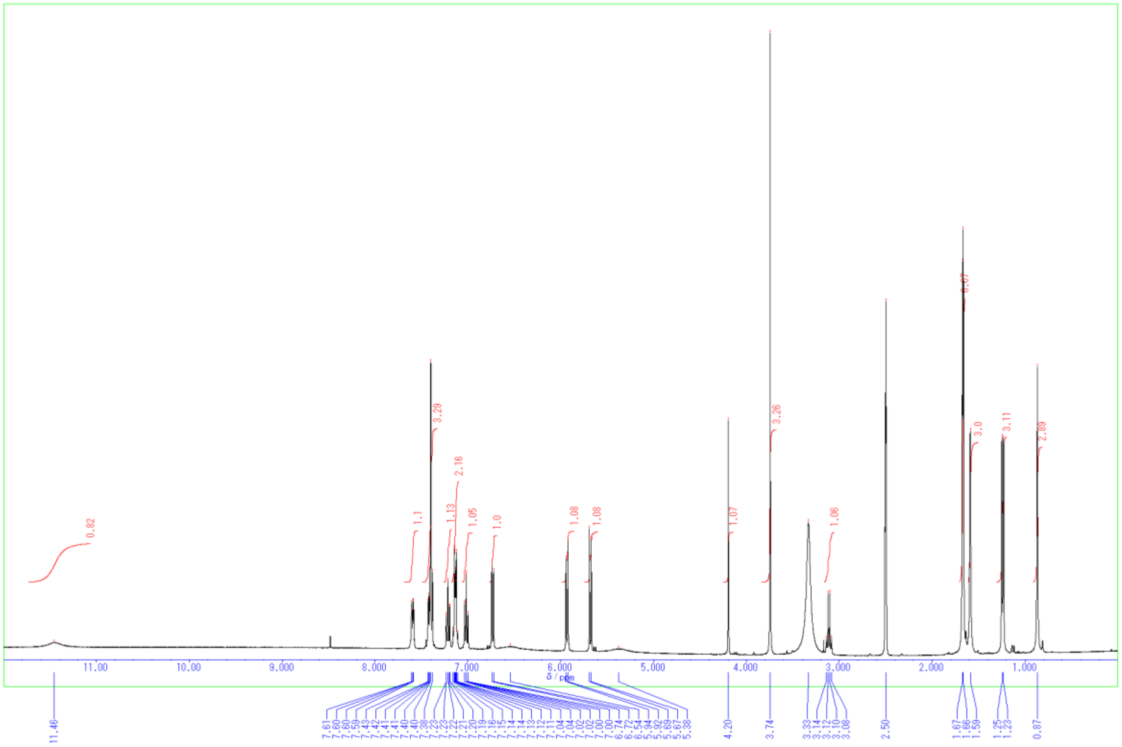

^{1}H-NMR of okaramine B in d6-DMSO

Supplementary Figure 1. ^{1}H- and ^{13}C NMR spectrum of okaramine B.

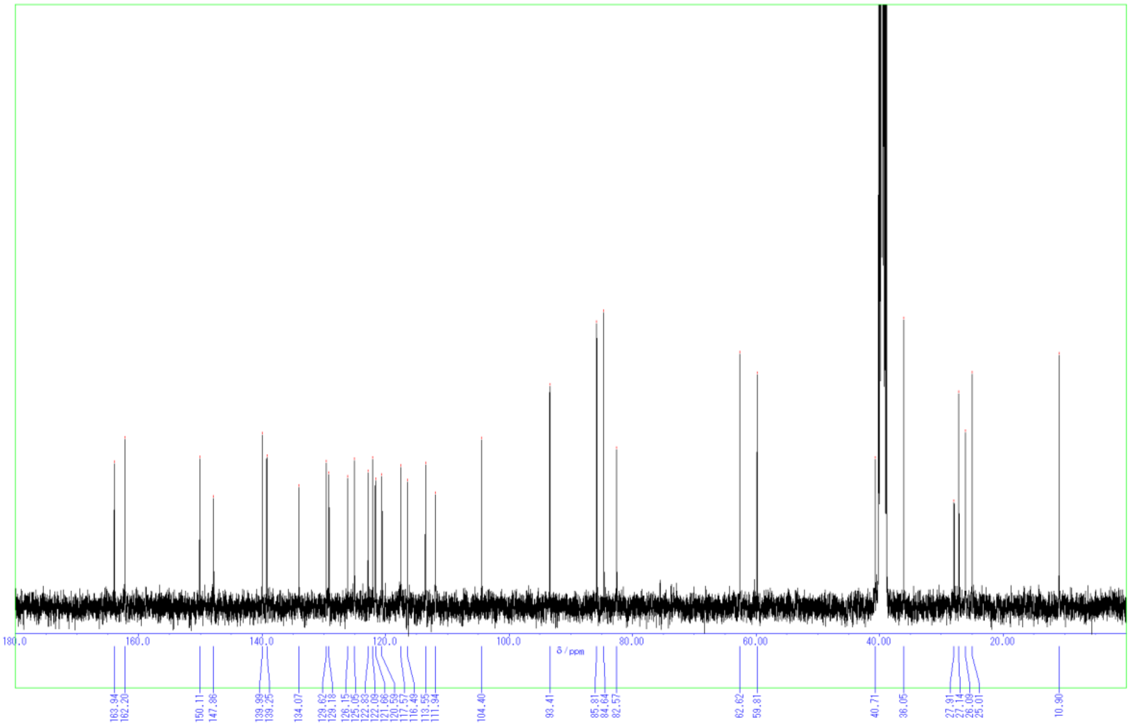

^{13}C-NMR of okaramine B in d6-DMSO

Supplementary Figure 1. continued

^{1}H-NMR spectral data for okaramine B

Observed[1,2]	Literature[1, 3]
0.87, 3H, s	0.86, 3H, s
1.24, 3H, d (7.6)	1.23, 3H, d (7.3)
1.59, 3H, s	1.58, 3H, s
1.66, 3H, s	1.65, 3H, s
1.67, 3H, s	1.66, 3H, s
3.10, 1H, q (7.6)	3.10, 1H, q (7.3)
3.74, 3H, s	3.73, 3H, s
4.20, 1H, s	4.19, 1H, s
5.36, 1H, br. s	5.26, 1H, s
5.68, 1H, d (8.2)	5.67, 1H, d (8.3)
5.93, 1H, d (8.2)	5.9, 1H, d (8.3)
6.53, 1H, br. s	6.42, 1H, s
6.73, 1H, d (7.8)	6.72, 1H, d (7.8)
7.03, 1H, td (7.8, 1.0)	7.01, 1H, t (7.8)
7.13, 1H, m	7.13, 1H, m
7.14, 1H, m	7.14, 1H, m
7.21, 1H, td (7.8, 1.3)	7.20, 1H, t (7.8)
7.39, 1H, d (7.8)	7.38, 1H, d (7.8)
7.40, 1H, s	7.39, 1H, s
7.39−7.44, 1H, m	7.41, 1H, d (4.4)
7.57−7.62, 1H, m	7.59, 1H, dd (5.4, 2.8)
11.46, 1H, br. s	11.42, 1H, br. s

[1]Chemical shifts in ppm, proton numbers, splitting and coupling constants (Hz).

[2]Long range couplings are taken into consideration to describe the splitting pattern.

[3]Data from Hayashi *et al.* (1989).

^{13}C-NMR spectral data for okaramine B

Observed[1]	Literature[1, 2]
10.9	11.1
25.0	25.2
26.1	26.3
27.1	27.3
27.9	28.1
36.1	36.2
40.7	40.9
59.8	60.1
62.6	62.3
82.6	82.7
84.6	84.8
85.8	86
93.4	93.6
104.	104.6
111.9	112.1
113.6	113.8
116.5	116.7
117.6	117.8
120.6	120.8
121.7	121.9
122.1	122.3
122.8	123
125.1	125.2
126.1	126.4
129.2	129.4
129.6	129.8
134.1	134.2
139.5	139.5
140.0	140.1
147.9	148.1
150.1	150.3
162.2	162.4
163.9	164.1

[1]Chemical shifts in ppm.

[2]Taken from Hayashi *et al.* (1989).

(A) TUAT-RH

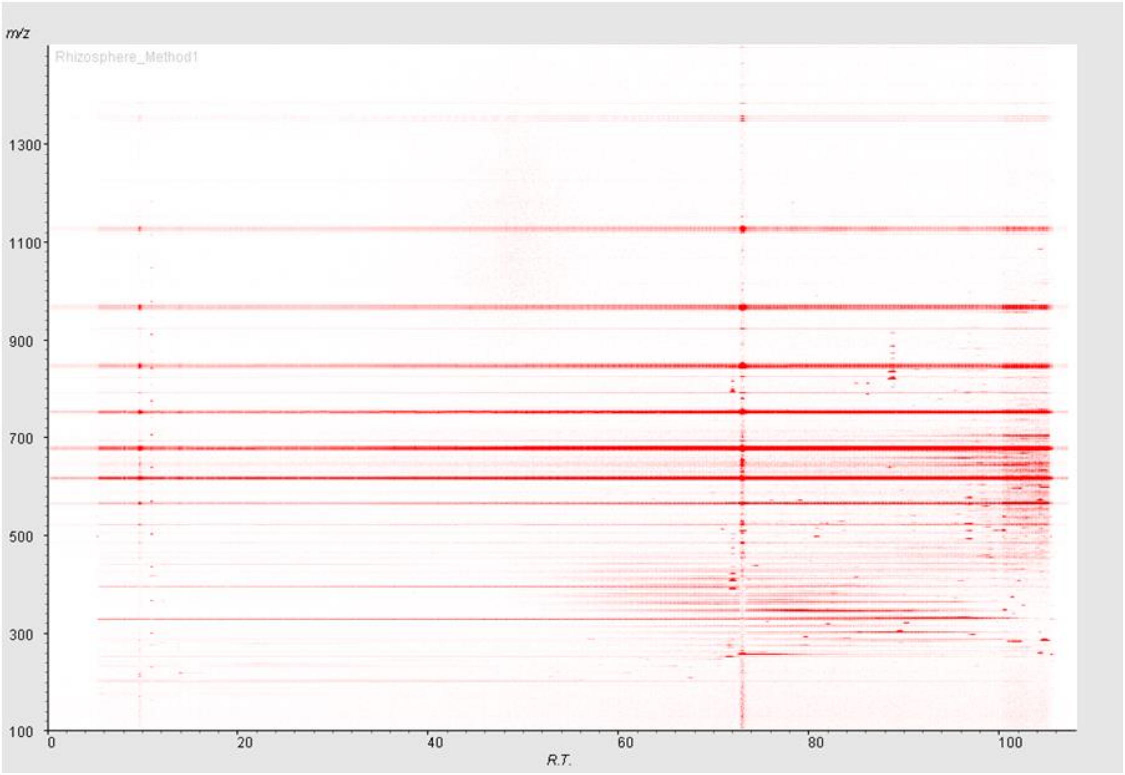

Supplementary Figure 2. Two-dimensional mass chromatogram obtained by untargeted metabolome analysis using LC-FT-ICR-MS. (A) TUAT-RH, (B) TUAT-RZ, (C) Kazusa, (D) KUAS-F, (E) KUAS-R, and (F) Blank control. The maximum color strength is based on a value of 1/32 of the maximum ion intensity in the mass chromatogram. X-axis: retention time (min). Y-axis: *m/z* value. A group of peaks eluted around 73 min was derived from the internal standard. Signals detected in all retention time are probably thought of as contaminants in the solvents.

(B) TUAT-RZ

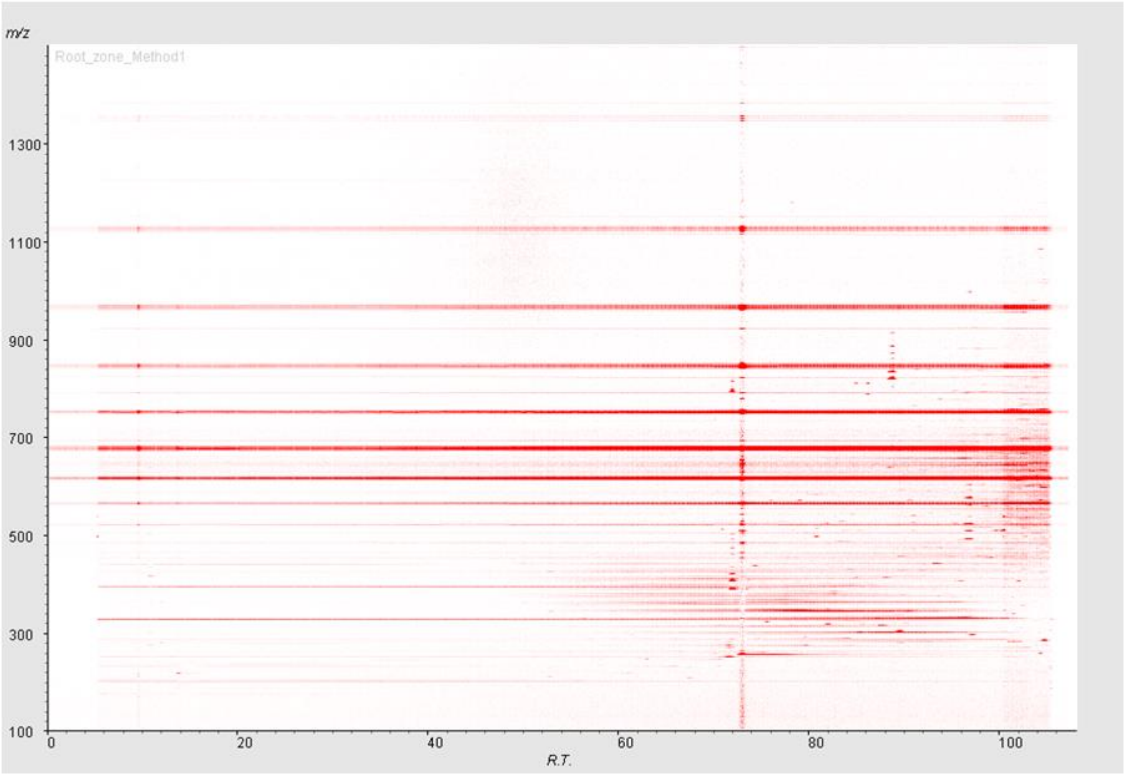

Supplementary Figure 2. Continued.

(C) Kazusa

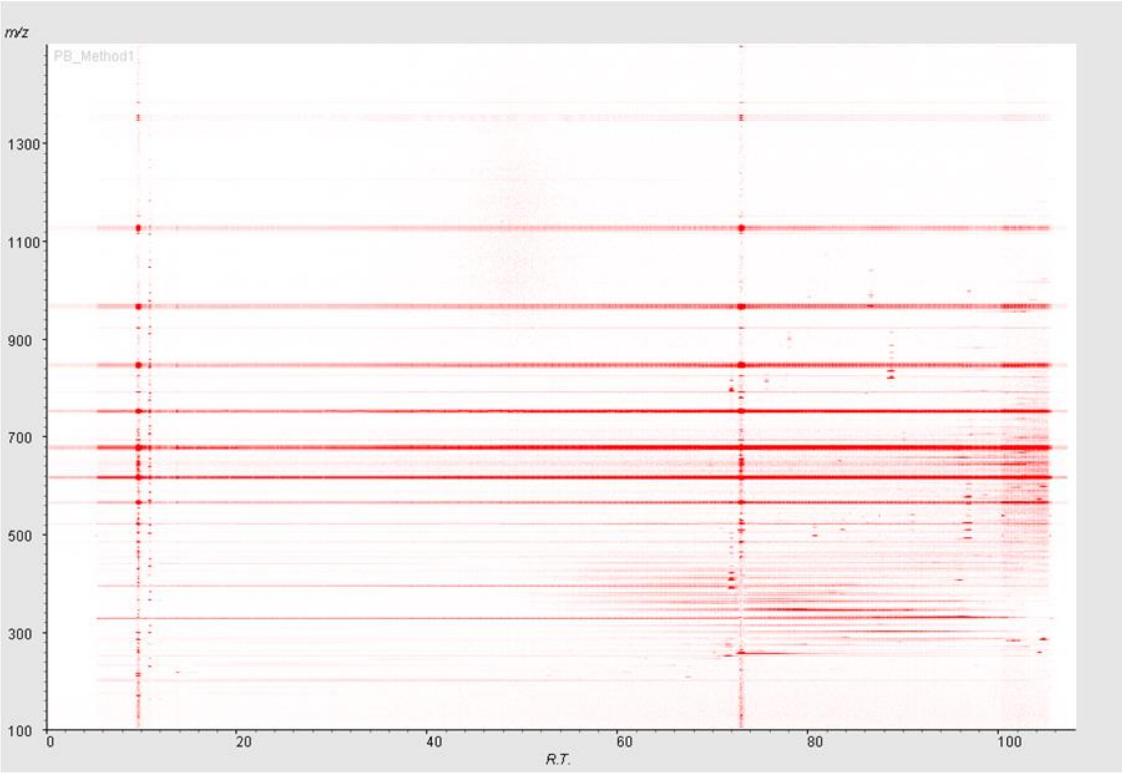

Supplementary Figure 2. Continued.

(D) KUAS-F

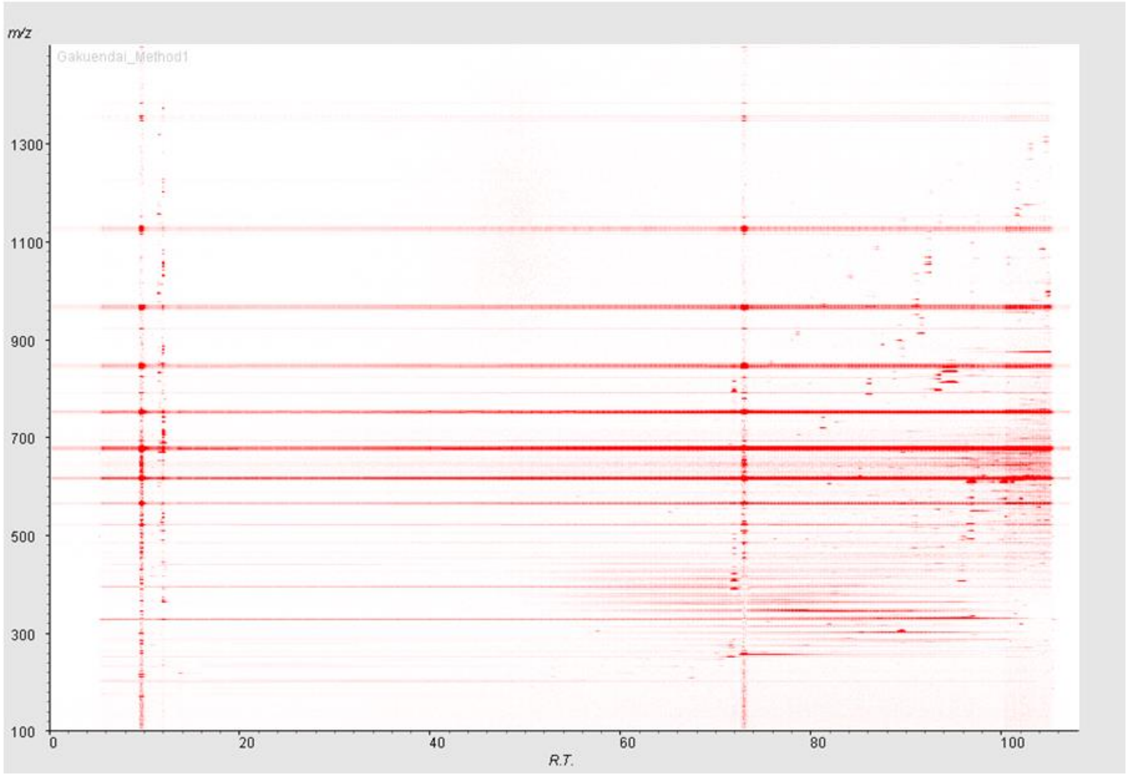

Supplementary Figure 2. Continued.

(E) KUAS-R

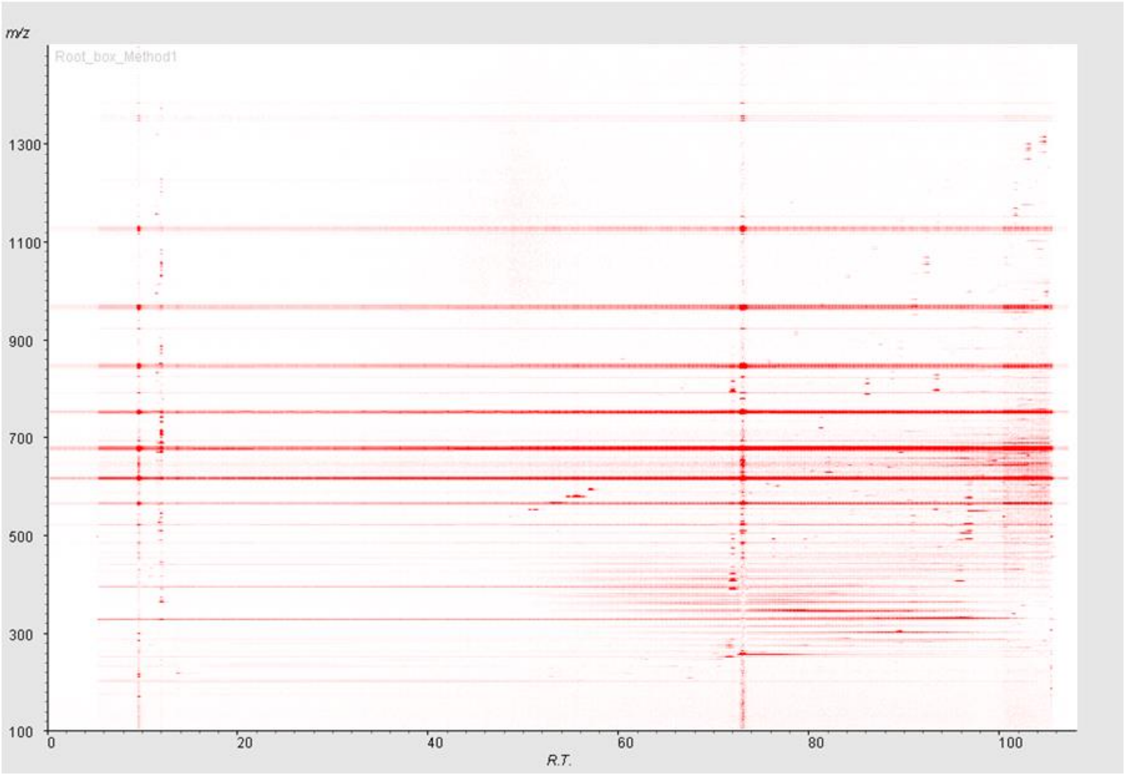

Supplementary Figure 2. Continued.

(F) Blank control

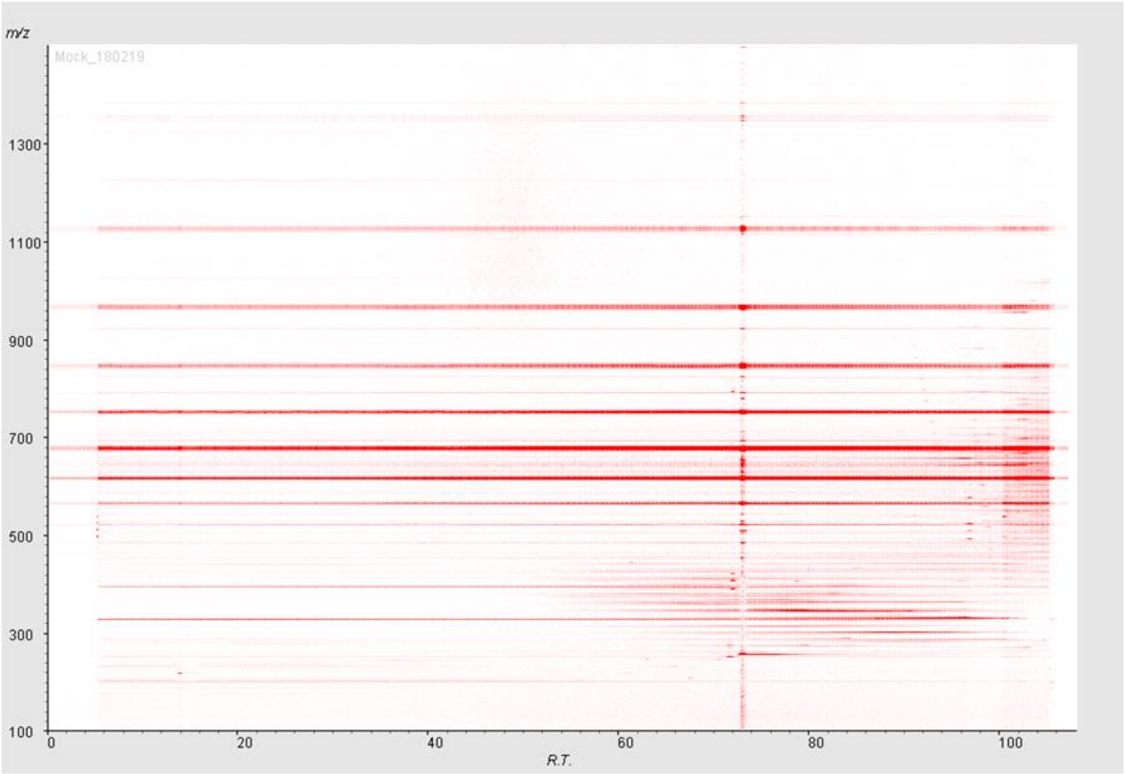

Supplementary Figure 2. Continued.

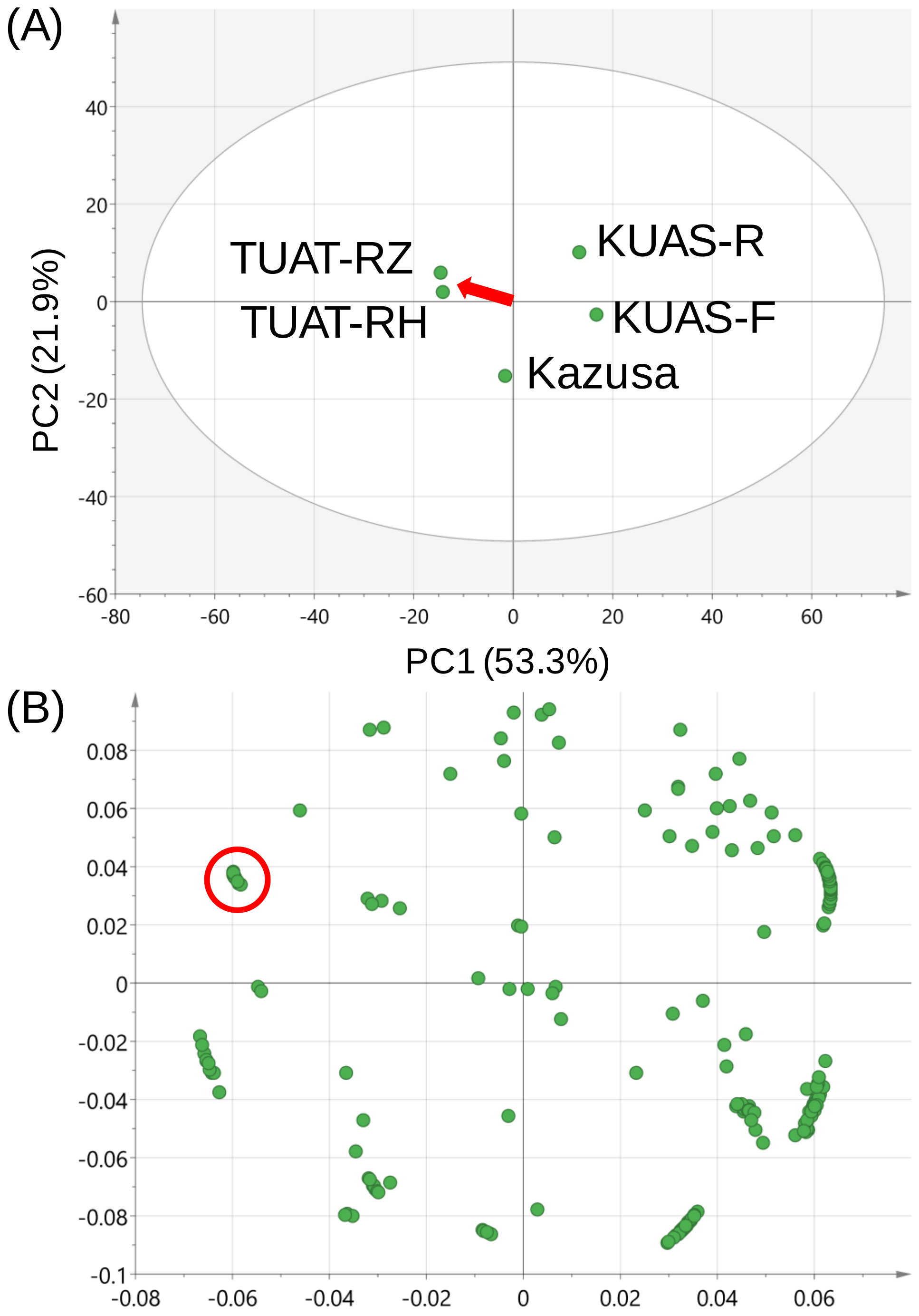

Supplementary Figure 3. Results of principal component analysis of soil metabolome data. (A) Score plot. (B) Loading plot. The red circle marks the location of 33 peaks which were specifically detected in the TUAT soils and absent in other soils. The arrow shows the direction from the origin to the location of the plot for TUAT soils. Exact loadings were described in Supplementary Tables 1 and 3.

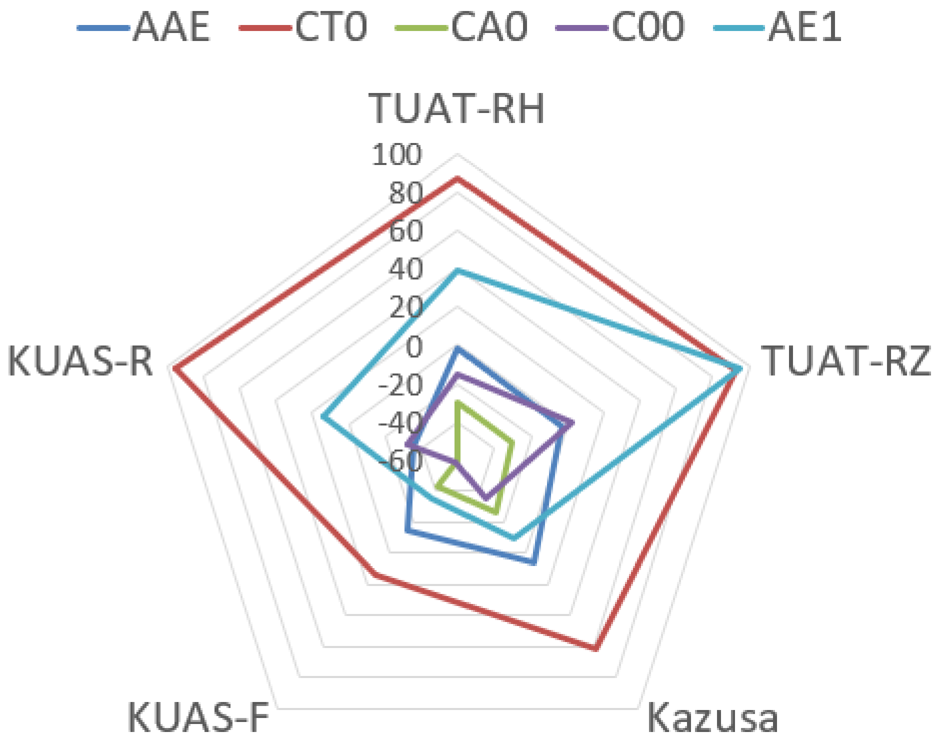

Supplementary Figure 4. Sensor output patterns of five soils given by the five indicated taste sensors. AAE for umami, CT0 for salty, CA0 for sour, C00 for bitter, AE1 for astringent.

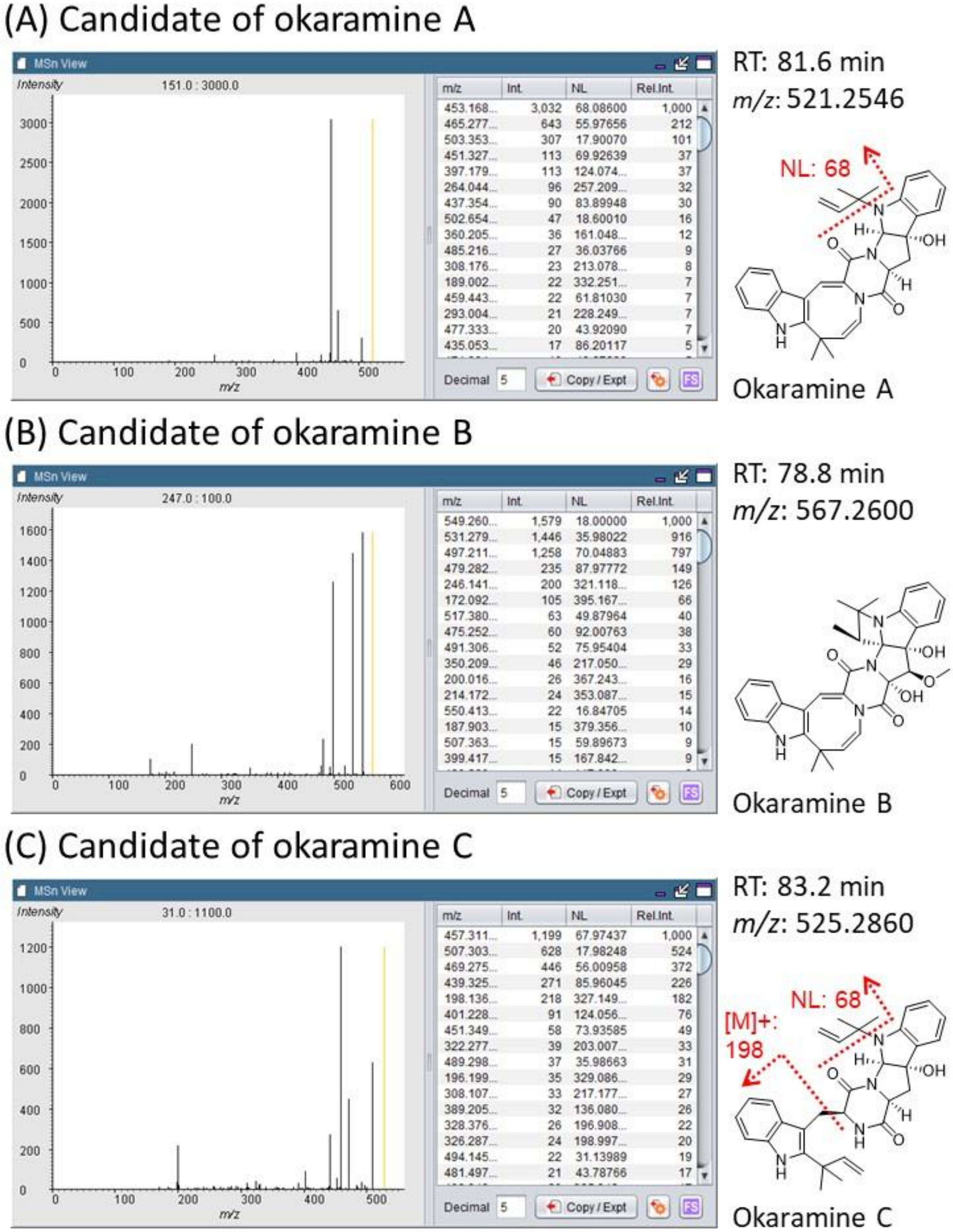

Supplementary Figure 5. MS^2 spectrum of the candidate peak of okaramine A (A), okaramine B (B), and okaramine C (C) in untargeted metabolome data from TUAT-RH obtained using LC-FT-ICR-MS. The neutral mass loss ("68") of the main product ion peak at *m/z* 453 in (A) and 457 in (C) can be attributed to the fragmentation of the C_5 structure in okaramines A and C, respectively.

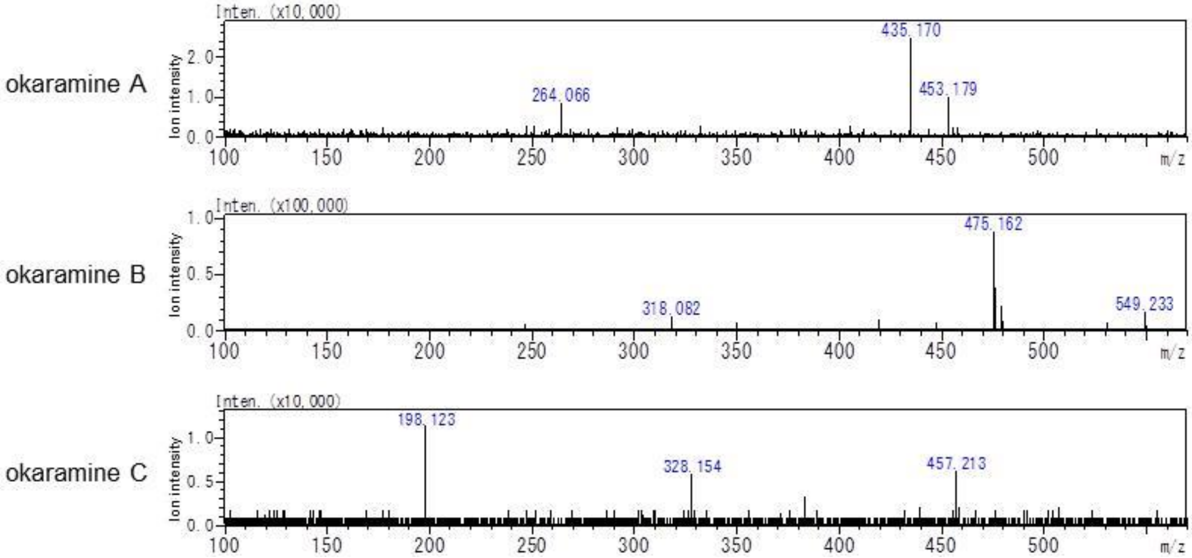

Supplementary Figure 6. MS/MS spectra for authentic standards of okaramines A, B, and C, and accurate mass of both authentic standards and TUAT soil samples obtained by LC-IT-TOF-MS.

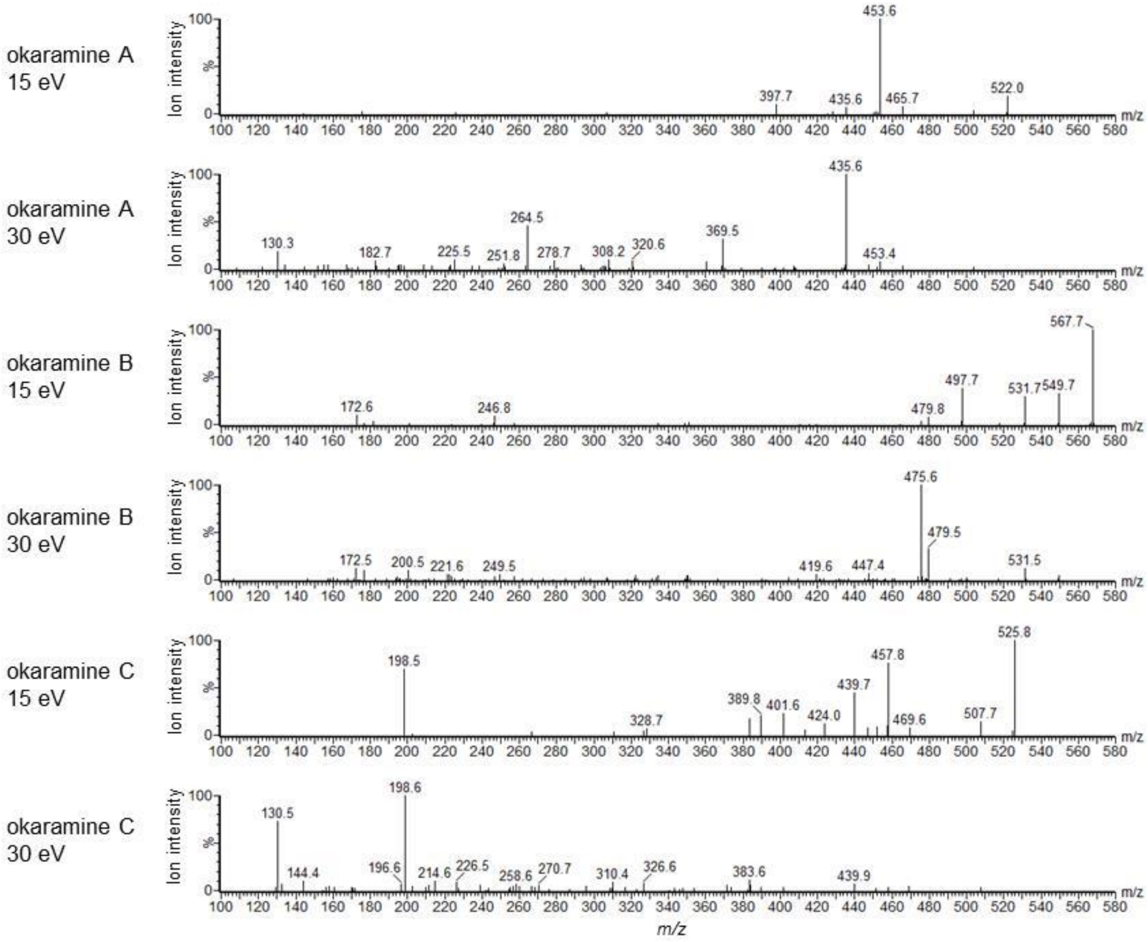

Supplementary Figure 7. MS/MS spectra of the authentic standards for okaramines A, B, and C obtained by LC-triple quadrupole MS with collision energies, 15 eV and 30 eV.

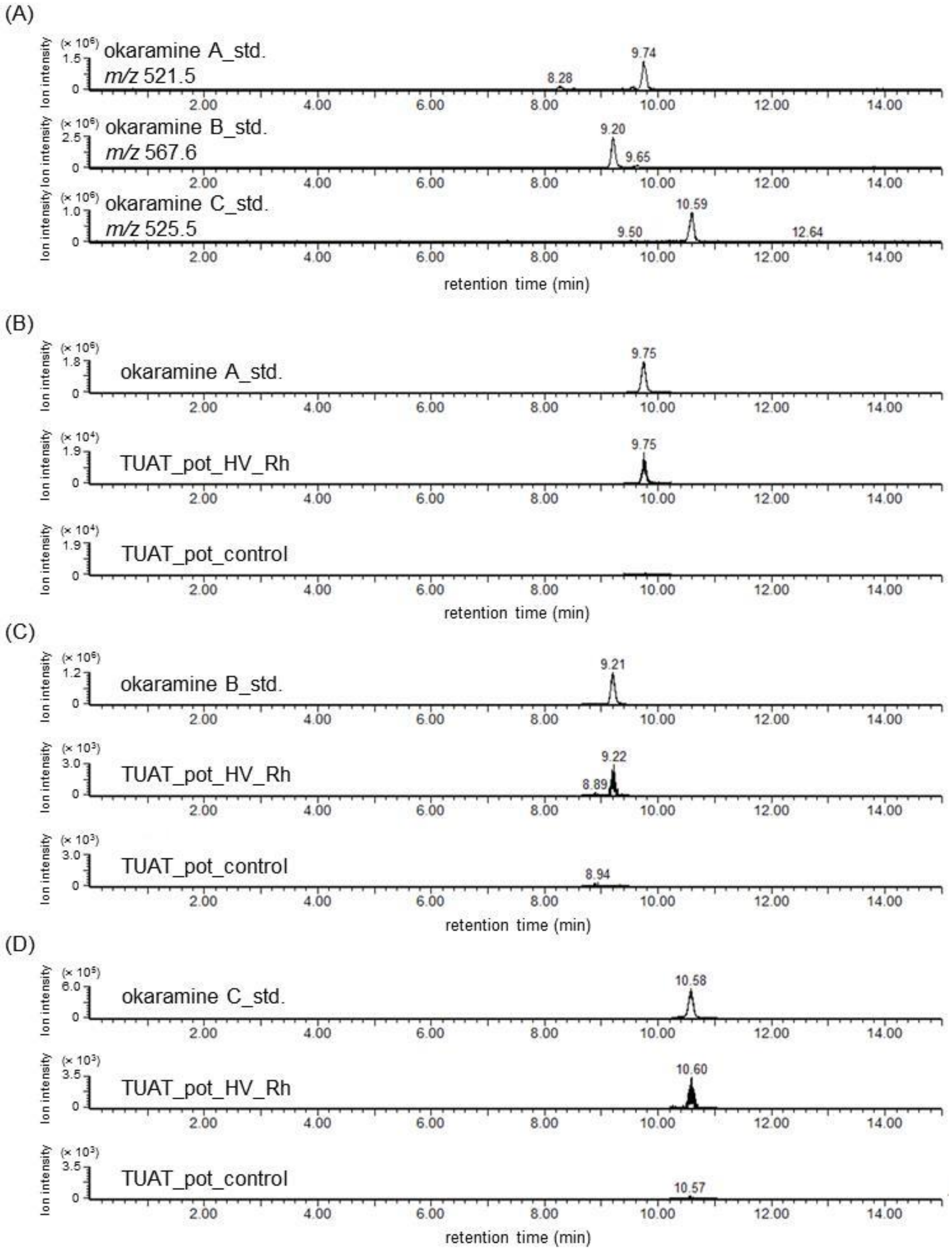

Supplementary Figure 8. LC-MS analysis of the authentic standards of okaramines A, B, and C, and extracts from rhizosphere soils of hairy vetch grown in pots using LC-triple quadrupole-MS. Extracted ion chromatograms for *m/z* 521.5, 567.6, and 525.5 of the authentic standards are shown in (A). Chromatograms obtained by MRM are shown for (B–D) okaramines A, B, and C. "HV_rhizo", rhizosphere soil of hairy vetch plot; "control" unplanted soil.

3 References

Arakawa, Y., Akagi, I., and Yamamoto, K. (2003). Determination of ammonium nitrogen in KCl extracts of cropland soils by using 2-hydroxybiphenyl sodium salt. *Jpn. J. Soil Sci. Plant Nutr.* 74(5)**,** 657-659. doi: 10.20710/dojo.74.5_657.

Nishiwaki, T., Mizukoshi, K., Ootake, N., and Ohyama, T. (1994). Determination of nitrate content in soybean by Cataldo's colorimetric method. *Jpn. J. Soil Sci. Plant Nutr.* 65(1)**,** 59-61. doi: 10.20710/dojo.65.1_59.

Tahara, Y., and Toko, K. (2013). Electronic tongues–A review. *IEEE Sensors J.* 13(8)**,** 3001-3011. doi: 10.1109/jsen.2013.2263125.

Toko, K., Tahara, Y., Habara, M., Kobayashi, Y., and Ikezaki, H. (2016). "Taste sensor: electronic tongue with global selectivity," in *Essentials of machine olfaction and taste,* ed. T. Nakamoto. (Singapore: John Wiley & Sons), 87-174.
